# Incidence of *Pneumocystis* Pneumonia in Immunocompromised Patients without Human Immunodeficiency Virus on Intravenous Pentamidine Prophylaxis: A Systematic Review and Meta-Analysis

**DOI:** 10.3390/jof9040406

**Published:** 2023-03-25

**Authors:** Chia-Yu Chiu, Patrick R. Ching

**Affiliations:** 1 Division of Infectious Diseases, Department of Internal Medicine, The University of Texas Health Science Center at Houston, Fannin St, Houston, TX 77030, USA; 2 Department of Infectious Diseases, Infection Control and Employee Health, The University of Texas MD Anderson Cancer Center, Houston, TX 77030, USA; 3 Division of Infectious Diseases, Department of Medicine, Washington University School of Medicine, St. Louis, MO 63110, USA; chingp@wustl.edu

**Keywords:** intravenous pentamidine, *Pneumocystis* pneumonia, prophylaxis, hematopoietic stem cell transplantation, immunocompromised host

## Abstract

Background: Trimethoprim-sulfamethoxazole (TMP-SMX) is a first-line *Pneumocystis* pneumonia (PCP) prophylaxis agent, but monthly intravenous pentamidine (IVP) is used in immunocompromised hosts without human immunodeficiency virus (HIV) infection because IVP is not associated with cytopenia and delayed engraftment. Method: We performed a systematic review and meta-analysis to estimate breakthrough PCP incidence and adverse reactions in HIV-uninfected immunocompromised patients receiving IVP. MEDLINE, Embase, Web of Science, Cochrane Library, and ClinicalTrials.gov were searched from their inception until 15 December 2022. Results: The pooled incidence of breakthrough PCP with IVP was 0.7% (95% CI, 0.3–1.4%, 16 studies, 3025 patients) and was similar when used as first-line prophylaxis (0.5%; 95% CI, 0.2–1.4%, 7 studies, 752 patients). The pooled incidence of adverse reactions was 11.3% (95% CI, 6.7–18.6%, 14 studies, 2068 patients). The pooled adverse event-related discontinuation was 3.7% (95% CI, 1.8–7.3%, 11 studies, 1802 patients), but was lower in patients receiving IVP monthly (2.0%; 95% CI 0.7–5.7%, 7 studies, 1182 patients). Conclusion: Monthly IVP is an appropriate second-line agent for PCP prophylaxis in certain non-HIV immunocompromised hosts, especially in patients with hematologic malignancies and hematopoietic stem cell transplant recipients. Using IVP for PCP prophylaxis as an alternative to oral TMP-SMX while patients are unable to tolerate enteral medication administration is feasible.

## 1. Introduction

In the absence of trimethoprim-sulfamethoxazole (TMP-SMX) prophylaxis, *Pneumocystis* pneumonia (PCP) develops in about 1% of immunocompromised hosts without human immunodeficiency virus (HIV) infection and has about a 1.8% PCP-related mortality [1]. TMP-SMX prophylaxis is highly efficacious in immunocompromised individuals without HIV, with a breakthrough PCP rate of about 0.2% [1]. TMP-SMX is the first-line agent for PCP prophylaxis in immunocompromised hosts [2,3,4]. The indication for PCP prophylaxis in immunocompromised hosts without HIV includes but is not limited to hematopoietic stem cell transplantation (HSCT) recipients (at least 6 months after allogenic HSCT and 3 months after autologous HSCT) [5], solid organ transplant (SOT) recipients (at least 6 months after transplantation) [4], and patients who took alemtuzumab (anti-CD52 monoclonal antibody), PI3K inhibitors, purine analogs, or other T-cell depleting agents (until CD4 count > 200 cells/mm^3^) [4,5]. However, patients with hematological malignancies, SOT recipients, or HSCT recipients might not be candidates for TMP-SMX because of hypersensitivity, bone marrow suppression, unstable renal function, or poor tolerance to oral medication.

Second-line agents for PCP prophylaxis including aerosolized pentamidine, dapsone, and atovaquone have been proposed despite lacking well-designed, properly randomized controlled trials [6]. Intravenous pentamidine (IVP) 4 mg/kg (maximum 300 mg) monthly is considered a second-line agent for the pediatric population in Europe but not in the United States [2,6]. Nevertheless, IVP is only approved for PCP treatment by the European Medicines Agency and the United States Food and Drug Administration [7,8,9]. IVP can be an alternative to dapsone or atovaquone in patients who cannot tolerate enteral administration. However, adverse reactions such as hypotension or hypoglycemia frequently occurred among persons living with HIV (PLHIV) in the 1990s when IVP was used, which raises concerns about the same side effects when used in individuals without HIV [10].

This meta-analysis aims to evaluate the breakthrough PCP incidence among immunocompromised individuals without HIV using IVP prophylaxis and its adverse reactions.

## 2. Methods

### 2.1. General Guidelines

This is a meta-analysis of event incidence. We followed the instructions of the latest version of the PRISMA 2020 guidelines (Supplementary Table S1) [11]. Ethics committee approval was not required because this is a systematic review of published data. This study was registered at: https://inplasy.com/inplasy-2022-12-0072/ (accessed on 18 December 2022, registered number: INPLASY2022120072).

### 2.2. Database Searches and Identification of Eligible Papers

Clinical studies reporting outcomes of IVP for PCP prophylaxis were screened. All study types except case reports and conference abstracts were considered. Studies that focused on PLHIV were excluded. MEDLINE, Embase, Web of Science, Cochrane Library, and ClinicalTrials.gov were searched from their inception to 15 December 2022 using the following search terms: (intravenous pentamidine) AND (*Pneumocystis jirovecii* pneumonia) AND (prophylaxis). The search strategy was applied to all databases. No language restrictions were applied to this search. Study eligibility was independently determined by two investigators (CC and PC), and differences were resolved by mutual consensus. The detailed search strategy for this systematic review and meta-analysis is provided in the Supplementary Materials (Supplementary Tables S2 and S3).

### 2.3. Data Extraction

All of the eligible articles were reviewed. The first author, year, sample size, number, prophylaxis characteristics, and participant characteristics were recorded. The incidence of breakthrough infection and adverse reactions to IVP were extracted from the published articles or provided by the authors upon request. The criteria for PCP were defined by each article. Breakthrough PCP was PCP that occurred while on prophylaxis. Primary prophylaxis was assigned to patients without a history of PCP. Secondary prophylaxis was assigned to patients who had a history of PCP to prevent recurrence. First-line prophylaxis was defined as IVP used as a first-line agent for PCP prophylaxis. Second-line prophylaxis was defined as IVP used when patients could not tolerate other PCP prophylaxis agents (TMP-SMX, dapsone, atovaquone, or aerosolized pentamidine) or when those agents were contraindicated. The pediatric population included patients aged younger than 18 years old, while the adult population included patients who were at least 18 years old. IVP monthly and IVP every 4 weeks were interchangeable during data extraction.

### 2.4. Quality Assessment

We adapted the risk of bias tool developed by Hoy et al. [12] and Edward et al. [13] to assess the overall quality of the available evidence on the incidence of breakthrough PCP and IVP adverse events. The tool includes nine items that assess measurement bias, selection bias, and bias related to the analysis (all rated as either low or high risk) and an overall assessment of risk of bias rated as either low, moderate, or high risk (Supplementary Tables S4 and S5).

### 2.5. Primary Outcome (Breakthrough Infection)

The primary outcome was breakthrough PCP in patients receiving IVP. The subgroup analysis and meta-regression included age (<18 years or ≥18 years), geographical region (study conducted in the United States or outside the United States), sample size (study patient size ≤ 100 or study patient size > 100), IVP monthly, and using IVP as first-line PCP prophylaxis. Breakthrough of *Toxoplasma* or *Nocardia* infection was also examined. 

### 2.6. Secondary Outcome (Adverse Reaction)

The secondary outcomes were the incidence of adverse reactions to IVP and IVP discontinuation due to adverse events. The subgroup analysis and meta-regression included age (<18 years or ≥18 years), geographical region (study conducted in the United States or outside the United States), sample size (study patient size ≤100 or >100), and IVP monthly.

### 2.7. Statistical Analysis

Based on the heterogeneous target populations in the recruited studies, the meta-analysis was conducted using a random-effects model. Between-trial heterogeneity was determined by using *I*^2^ tests; an *I*^2^ > 50% was considered statistically significant heterogeneity. Funnel plots and Egger’s test were used to examine potential publication bias. The level of significance was 5%. For cells with zero-event, the zero was replaced by 0.5 to enable software calculation to properly include the study in the analysis [14]. All analyses were performed using Comprehensive Meta-Analysis (CMA) software, version 3.3 (Biostat, Englewood, NJ, USA).

## 3. Results

### 3.1. Study Selection

**Figure 1:**
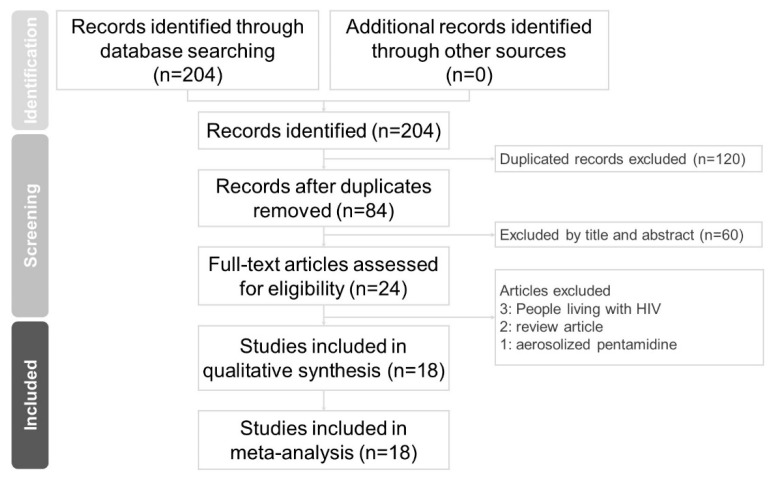
PRISMA flow diagram for study selection.

The PRISMA flow chart of the literature search process is presented in Figure 1. After removing the duplicate articles and excluding non-relevant articles by reading the titles and abstracts, 18 articles (3115 patients) were included in this meta-analysis. The characteristics of the studies included are summarized in Table 1. Among these 18 articles, 17 are retrospective design (3065 patients) and 1 is a prospective observational design (50 patients). Among these 18 articles, 11 articles included pediatric populations (1874 patients), 5 articles included adult populations (1151 patients), and 2 articles examined patients in all age groups (90 patients). Thirteen studies administrated IVP monthly exclusively (2378 patients). Seven articles (775 patients) used premedication (including antiemetics, antihistamine, or benzodiazepine) before IVP. All these articles were published after 2008.

### 3.2. Methodological Quality of the Included Studies

Six studies had a moderate risk of bias and twelve studies had a low risk of bias. The main risk bias came from (1) the definition of PCP and (2) the retrospective study design. The detailed risk of bias evaluation is summarized in the Supplementary Materials (Supplementary Tables S4 and S5).

#### 3.2.1. Primary Outcome: Breakthrough Incidence of PCP on IVP

Sixteen studies (3025 patients) reported the breakthrough incidence of PCP, including five studies in adult populations and eleven studies in pediatric populations. Among these 16 studies, 5 studies used IVP as first-line PCP prophylaxis, 3 studies used IVP as either first- or second-line prophylaxis, and 8 studies used IVP as second-line prophylaxis. In adult populations, underlying conditions included hematologic malignancies, autologous HSCT, and allogeneic HSCT. In pediatric populations, underlying conditions included solid tumors, hematologic malignancies, autologous HSCT, allogeneic HSCT, and SOT. The pooled breakthrough PCP was 0.7% (95% confidence interval [CI] 0.3–1.4%, 16 studies, 3025 patients) (Supplementary Figures S1A and S2A).

This breakthrough rate did not vary whether the studies were performed in or outside of the United States (0.7%, 95% CI 0.3–1.6%, 13 studies, 2677 patients vs. 0.7%, 95% CI 0.2–2.7%, 3 studies, 348 patients) (Supplementary Table S6). The breakthrough PCP was lower in the adult populations (0.3%, 95% CI 0.1–1.1%, 5 studies, 1151 patients) than in the pediatric populations (0.8%, 95% CI 0.3–2.1%, 11 studies, 1874 patients). The breakthrough PCP was lower in studies with >100 patients (0.5%, 95% CI 0.3–1.0%, 12 studies, 2809 patients) than in those with ≤100 patients (2.1%, 95% CI 0.3–14.2%, 4 studies, 216 patients). The breakthrough PCP was 0.7% (95% CI 0.2–2.0%, 11 studies, 2288 patients) in patients receiving IVP monthly and 0.5% (95% CI 0.2–1.4%, 7 studies, 752 patients) in patients receiving IVP as a first-line agent. In the multivariate meta-regression, age group (β = 1.956, 95% CI 0.494–3.418, *p* = 0.009) and total patient number (β = 2.249, 95% CI 0.956–3.542, *p* = 0.001) were significantly associated with the incidence of breakthrough PCP (Supplementary Table S7). 

#### 3.2.2. Primary Outcome: Breakthrough Toxoplasma or Nocardia Infection on Intravenous Pentamidine

Three studies (1222 patients) reported 0.5–0.7% breakthrough for *Toxoplasma* [18,19,24]. One study (702 patients) reported 1% breakthrough for *Nocardia* [19].

#### 3.2.3. Secondary Outcome: Incidence of Adverse Reaction in Patients Receiving Intravenous Pentamidine

Fourteen studies (2068 patients) reported incidence of adverse reactions, including nine studies in pediatric populations, three studies in adult populations, and two studies in mixed populations (adult and pediatric). The pooled incidence of adverse reactions was 11.3% (95% CI, 6.7–18.6%, 14 studies, 2068 patients) (Supplementary Figures S1B and S2B).

The incidence of adverse reactions was lower in the adult populations (2.7%, 95% CI 0.1–51.8%, 3 studies, 336 patients) than in the pediatric populations (9.5%, 95% CI 5.9–14.9%, 9 studies, 1642 patients), lower in studies performed outside the United States (4.8%, 95% CI 0.8–23.6%, 3 studies, 360 patients) than in the United States (13.2%, 95% CI 7.3–22.8%, 11 studies, 1708 patients), and lower in studies with >100 patients (8.6%, 95% CI 5.1–14.2%, 9 studies, 1774 patients) than those with ≤100 patients (18.9%, 95% CI 4.7–52.5%, 5 studies, 294 patients). The incidence of adverse reactions was 12.3% (95% CI 5.3–25.7%, 9 studies, 1331 patients) in patients who used IVP monthly (Supplementary Table S8). These covariates were not statistically significant in multivariable meta-regression model (Supplementary Table S9).

#### 3.2.4. Secondary Outcome: Discontinuation of Intravenous Pentamidine Due to Adverse Events

A total of 11 studies (1802 patients) reported adverse reaction-related discontinuation including 8 studies in pediatric populations, 1 study in an adult population, and 2 studies in mixed (adult and pediatric) populations. The pooled discontinuation rate was 3.7% (95% CI, 1.8–7.3%, 11 studies, 1802 patients) (Supplementary Figures S1C and S2C).

The adverse event-related discontinuation was lower in studies performed outside the United States (1.8%, 95% CI 0.6–5.5%, 3 studies, 360 patients) than those performed in the United States (4.7%, 95% CI 2.1–9.9%, 8 studies, 1442 patients), and lower in studies with >100 patients (3.0%, 95% CI 1.2–6.9%, 8 studies, 1657 patients) than those with ≤100 patients (6.6%, 95% CI 1.9–20.2%, 3 studies, 145 patients). The adverse event-related discontinuation (0.2%, 95% CI 0.7–5.7%, 7 studies, 1182 patients) in patients receiving IVP monthly was lower than pooled discontinuation (3.7%, 95% CI 1.8–7.3%, 11 studies, 1802 patients) (Supplementary Table S10). In the multivariate meta-regression, the frequency of IVP (β = 1.800, 95% CI 0.265–3.335, *p* = 0.022) was significantly associated with adverse event-related discontinuation (Supplementary Table S11).

Common adverse events including nausea, vomiting, and paresthesia seldom led to IVP discontinuation (Table 1). Two of the patients experienced cardiac arrest due to rapid infusion (<60 min) [23]. 

## 4. Discussion

To the best of our knowledge, this is the first meta-analysis that evaluates IVP with PCP breakthrough in immunocompromised hosts without HIV. Our findings suggest that using IVP for PCP prophylaxis as an alternative to oral TMP-SMX while patients are unable to tolerate enteral medication administration is feasible.

A direct comparison between IVP and TMP-SMX in PCP prophylaxis is lacking. However, comparing the results of our study (focusing on IVP) and a published meta-analysis (focusing on TMP-SMX) [1], the pooled breakthrough PCP was higher in IVP than in TMP-SMX (0.7% vs. 0.2%) in immunocompromised patients without HIV. This finding is consistent with the current guidelines state that TMP-SMX should be the first-line agent for PCP prophylaxis [3,6].

TMP-SMX is also an effective prophylaxis agent for *Toxoplasma* reactivation [3]. A preemptive approach with weekly monitoring of blood *Toxoplasma* PCR is a reasonable option in these populations if TMP-SMX prophylaxis cannot be used [33]. Breakthrough *Nocardia* infection is 1% in allogeneic HSCT recipients receiving IVP for PCP prophylaxis [19]. High-dose TMP-SMX (15 mg/kg IV of the TMP component) is the recommended treatment for *Nocardia* infection, but low-dose TMP-SMX (160/800 mg daily or three times/week) is not effective in preventing *Nocardia* infection in immunocompromised hosts [34,35]. Since low-dose TMP-SMX does not prevent *Nocardia* infections in immunocompromised hosts, concern regarding IVP having a higher breakthrough *Nocardia* infection than low-dose TMP-SMX is not justifiable and should not be used as a reason against using IVP as an alternative to TMP-SMX for PCP prophylaxis.

The utility of IVP for PCP prophylaxis in immunocompromised hosts without HIV is extrapolated from the experience in PLHIV. In PLHIV, primary or secondary PCP prophylaxis with IVP every two weeks (Q2W), every three weeks (Q3W), or monthly has been used [10,36,37,38,39]. PCP prophylaxis in PLHIV has a breakthrough rate of 0–28.5% when using IVP monthly and 5.5% when using IVP Q2W [40]. The pharmacokinetic study demonstrated 10–14 days of IVP elimination half-life, suggesting that monthly dosing may be inferior to more frequent dosing for PCP prophylaxis [41]. In addition, a human autopsy study revealed that pentamidine levels remained detectable in the lung tissue for a few weeks after the last dose, but the low lung tissue concentration (less than therapeutic level 30 μg/g) raised concerns about poor efficacy [42]. Based on the above data, some clinicians use IVP Q2W or IVP Q3W for PCP prophylaxis, particularly in age < 2 years, because higher breakthrough PCP with IVP monthly has been observed in this subgroup [26]. We found that IVP Q2W or Q3W were used in pediatric populations (4 out of 11 articles). In adults, all patients (5 out of 5 articles) received IVP monthly. The pooled breakthrough PCP rate (0.7%) was no different than the rate for patients receiving IVP monthly (0.7%). On the other hand, compared with the pooled data (the combination of patients on Q2W, Q3W, and monthly dosing), patients on IVP monthly had a similar incidence of adverse reactions (11.3% vs. 12.3%) but a lower rate of adverse events-related discontinuation (3.7% vs. 2.0%; multivariate meta-regression *p* = 0.022). Although studies directly comparing monthly and more frequent IVP administration (Q2W or Q3W) are not available, IVP monthly appears to be effective and safe based on the above data.

Common adverse reactions to IVP include nausea, vomiting, tingling, numbness, and unpleasant taste. These adverse reactions are typically mild, self-limited, or easy to control by premedication. They start early during transfusion and tend to be recurrent [17,18,32,38]. However, anaphylaxis, transfusion rate-related hypotension, dysglycemia (hypoglycemia or hyperglycemia), and pancreatitis are worrisome adverse reactions that usually lead to IVP discontinuation [40]. Premedication with antiemetic, antihistamine, antipyretic, or benzodiazepine might decrease the incidence or severity of adverse reactions and improve patients’ tolerance of IVP [16,18,22,25]. In one study among PLHIV, normal saline bolus prior to IVP showed a reduced drop in blood pressure (defined as mean blood pressure decrease >10 mmHg), but it did not reach a statistical difference [38]. Rapid infusion (<60 min) of IVP can cause hypotension and arrhythmia. In our review, two patients experienced cardiac arrest by erroneous rapid infusion [23]. IVP is administered for 1–2 h in common practice (Table 1). Clinicians need to be aware that rapid IVP infusion can lead to cardiac arrhythmia.

Aerosolized pentamidine (AP) was used for PCP prophylaxis because it directly delivers the drug to the alveoli and reduces the systemic toxicity noted in IVP. Breakthrough PCP has been reported in 5–25% of patients receiving AP in PLHIV [40]. In PLHIV, AP has similar efficacy and tolerability for PCP prophylaxis in comparison to IVP [36], but it is less successful than IVP for severe PCP treatment [43]. However, there is no comparison of efficacy between AP and IVP for immunocompromised hosts without HIV on PCP prophylaxis or PCP treatment. Some retrospective studies have evaluated tolerance of immunocompromised patients without HIV to AP and IVP, but the results have been controversial. Two studies favor AP [23,31] and the other two favor IVP [30,36]. 

Caspofungin has in vivo efficacy in treating PCP when used solely or combined with TMP-SMX [44]. Rezafungin, a novel echinocandin administered intravenously once weekly, has shown efficacy of PCP prophylaxis in mouse models [45]. Currently, a prospective, multicenter, randomized trial (NCT04368559) examining the efficacy of rezafungin for PCP prophylaxis may provide another intravenous option for PCP prophylaxis in the future.

There are several limitations of this meta-analysis. Firstly, the incidence of IVP adverse reactions may be underestimated in the retrospective chart review because mild symptoms may not be documented. Secondly, in the subgroup analysis, we compared the frequency of IVP dosing between the pooled (the combination of Q2W, Q3W, and monthly) and monthly only. The existing data that combined the outcomes for Q2W and Q3W, or Q3W and monthly, did not allow for us to perform an analysis of IVP, Q2W, and Q3W individually. Thirdly, a subgroup analysis based on the patients’ underlying conditions (solid tumors, hematologic malignancies, autologous HSCT, allogeneic HSCT, and SOT) was not performed because of the limited information from the original data. Fourthly, the efficacy of IVP in other immunocompromised hosts (such as patients with autoimmune disorders taking immunosuppressants or receiving a glucocorticoid dose equivalent to ≥20 mg of prednisone daily for one month) could not be assessed because no studies were identified. Fifthly, many patients received TMP-SMX as a first-line prophylaxis before IVP and were switched back to TMP-SMX once they were able to. The breakthrough PCP may have been confounded by TMP-SMX use during the follow-up period. Similarly, confounding results may also be present for patients who first received IVP and then switched to TMP-SMX once they were able to tolerate oral medication. Sixthly, for the literature supporting PCP prophylaxis in immunocompromised patients without HIV, the majority come from pediatric populations. In this meta-analysis, the PCP breakthrough rate (primary outcome) is lower in adult populations (0.3%, 5 studies) than in pediatric populations (0.8%, 11 studies), but the incidence of adverse events or adverse event-related discontinuation (secondary outcome) is mainly derived from pediatric populations (9 out of 14 articles; 8 out of 11 articles). More clinical research is warranted in adult populations. 

## 5. Conclusions

In conclusion, IVP monthly is an appropriate second-line agent for PCP prophylaxis in certain immunocompromised hosts without HIV, especially in hematologic malignancies, and HSCT. Using IVP when oral TMP-SMX could not be given temporarily and switching to TMP-SMX when patients is able to tolerate oral medication is a safe practice. The incidence of adverse events seem to be low, and patients usually tolerate IVP with premedication. However, larger prospective studies should be conducted to validate efficacy and tolerability in immunocompromised hosts without HIV receiving IVP.

## Figures and Tables

**Table 1:** Studies of patients who received intravenous pentamidine for PCP prophylaxis.

Author, Country, Year, Reference	Study Type, Study Period	Patient Number, Characteristics ^a^	Frequency, Infusion Rate, Premedication	Breakthrough Incidence	Adverse Reactions
**Adult, receiving chemotherapy or HSCT**					
Lim, USA, 2015, [15]	Retrospective, January 2011–December 2013	99 allo HSCT; primary prophylaxis; first-line prophylaxis	Q4W; NA; NA.	PCP: 0/99 (0%)	0/99 (0%)
Diri, USA, 2016, [16]	Retrospective, January 2001–May 2013	113 allo HSCT; 74 primary prophylaxis, 39 secondary prophylaxis; first-line prophylaxis	Q4W (until the patient can tolerate oral medication); NA; diphenhydramine, ondansetron	PCP: 0/113 (0%)	NA
Sweiss, USA, 2018, [17]	Prospective, March 2015–June 2016	24 chemotherapy, 16 auto HSCT, 10 allo HSCT; primary prophylaxis; first-line prophylaxis	Q4W; 2 h; Ondansetron	PCP: 0/50 (0%)	17/50 (34%). Hypotension (n = 6), nausea (n = 4), nasal congestion (n = 2), oral numbness (n = 2), and rash (n = 1). No adverse event-related discontinuation.
Awad, Jorden, 2020, [18]	Retrospective, January 2014–September 2018	65 auto HSCT, 122 allo HSCT; first-line prophylaxis or second-line prophylaxis	Q4W; 1 h; ranitidine, hyoscine, and/or metoclopramide	PCP: 0/187 (0%) *Toxoplasma*: 1/187 (0.5%)	1/187 (0.5%) patients. No adverse event-related discontinuation.
McCollam, USA, 2022, [19]	Retrospective, January 2007–September 2017	702 allo HSCT; primary prophylaxis or secondary prophylaxis; second-line prophylaxis	Q4W (until can tolerate oral TMP-SMX) NA; NA	PCP: 0/702 (0%); *Toxoplasma*: 5/702 (0.7%); *Nocardia*: 7/702 (1%)	Only 280 patients’ charts were available. Nausea (n = 47) and generalized pain/discomfort (n = 13). Other adverse events (n ≤ 4), indigestion, diarrhea, numbness, hypotension, rash, dizziness, tachycardia, hypomagnesemia, and weakness.
**Pediatric, malignancy (solid tumors and hematologic malignancies) or transplantation (HSCT and SOT)**					
Kim, USA, 2008, [20]	Retrospective, January 2001–May 2006	232 cancer patients; second-line prophylaxis	Q4W; 2 h; NA	PCP: 3/232 (1.3%), 2/106 (1.9%) in HSCT subgroup.	NA
Prasad, USA, 2008, [21]	Retrospective, June 2003–June 2005	12 cancer patients; second-line prophylaxis	Q4W, NA; NA	PCP: 2/12 (16.7%)	NA
DeMasi, USA, 2013, [22]	Retrospective, January 2005–October 2011	137 patients with 167 HSCT events (113 auto HSCT, 54 allo HSCT); Primary prophylaxis; First line prophylaxis	Q4W; 1 h; antiemetics	PCP: 0/137 (0%)	12/137 (8.8%). Nausea/vomiting (n = 10), and anaphylaxis (n = 2). Two patients discontinued IVP because of adverse events.
Orgel, USA, 2014, [23]	Retrospective, 2005–2010	117 cancer patients; second-line prophylaxis	NA; NA; NA	PCP: 1/117 (0.9%)	10/117 (8.5%). Two patients experienced cardiac arrest after rapid infusion (<60 min).
Clark, USA, 2015, [24]	Retrospective, January 2010–July 2013	287 HSCT, 46 SOT; second-line prophylaxis	Q3W or Q4W; NA; NA	PCP: 1/333 (0.3%) *toxoplasma*: 2/333 (0.6%)	20/333 (6%) ^b^ Tachycardia (n = 7), shortness of breath (n = 4), pancreatitis (n = 2), QTc prolongation (n = 2), elevated LFT (n = 2), fever (n = 1), numbness (n = 1), and anaphylaxis (n = 1). Twenty patients discontinued IVP because of adverse events.
Curi, USA, 2016, [25]	Retrospective, January 2007–December 2012	142 allo HSCT; first-line prophylaxis	Q4W; 2 h; diphenhydramine, acetaminophen, ondansetron and/or lorazepam	PCP: 0/142 (0%)	29/142 (20.4%). Paresthesia (n = 6), headache (n = 2), nasal congestion (n = 2), cough (n = 2), dyspnea (n = 2), nausea (n = 2), vomiting (n = 2), hives (n = 2), agitation (n = 1), lightheadedness (n = 1), throat itchiness (n = 1), hypertension (n = 1), hypotension (n = 1), lip swelling (n = 1), flushing (n = 1), and non-specified rash (n = 1). No adverse events-related discontinuation.
Levy, USA, 2016, [26]	Retrospective, December 2006–June 2013	111 patients with 141 HSCT events (27 auto HSCT, 114 allo HSCT); second-line prophylaxis	Q2W; NA; NA	PCP: 0/111 (0%)	14/111 (12.6%) Hypotension (n = 3), abdominal pain/nausea/vomiting (n = 2), pancreatic dysfunction (n = 3), rash/pruritus (n = 2), perioral numbness/tingling (n = 2), dyspnea/tachycardia (n = 1), hepatotoxicity (n = 1), and nephrotoxicity (n = 1). Twenty-one patients discontinued IVP because of adverse events.
Solodokin, USA, 2016, [27]	Retrospective, January 2009–July 2014	121 cancer patients (12 auto HSCT, 4 allo HSCT); primary prophylaxis or secondary prophylaxis; first-line prophylaxis or second-line prophylaxis	Q3W or Q4W; NA; Yes, but regimen is not available	PCP: 0/121 (0%)	19/121 (15.7%). Allergic reaction (n = 6), nausea (n = 5), facial paresthesia (n = 4), hypotension (n = 4), perioral numbness (n = 3), and extravasation (n = 1). Five patients discontinued IVP because of adverse events.
Tamyao, Spain, 2017, [28]	Retrospective, March 2007–January 2017	55 patients with 92 auto HSCT events; second-line prophylaxis	Q2W or Q3W; 1 h; NA	PCP: 0/55 (0%)	3/55 (5.5%). Anaphylaxis (n = 1), seizure (n = 1), and nausea/hypotension (n = 1). Two patients discontinued IVP because of adverse events.
Kruizinga, Netherland, 2017, [29]	Retrospective, May 2011–September 2016	106 cancer patients; first-line prophylaxis or second-line prophylaxis	Q4W; 1 h; NA	PCP: 1/106 (0.9%)	21/118 (17.8%) courses. Nausea (n = 14), tachycardia (n = 3), dyspnea (n = 3), rash/itch (n = 3), hypotension (n = 2), fever (n = 2), and paresthesia (n = 2). Two patients discontinued IVP because of adverse events.
Quinn, USA, 2018, [30]	Retrospective, January 2007–August 2014	508 cancer patients; second-line prophylaxis	Q4W, 1 h; NA	PCP: 0/508 (0%)	11/508 (2.2%) ^b^ Anaphylaxis (n = 4), hypotension (n = 3), tingling/numbness (n = 2), pancreatitis (n = 1), and dyspnea (n = 1). Eleven patients discontinued IVP because of adverse events.
**Mixed with pediatric and adult patients**					
Brown, USA, 2020, [31]	Retrospective, January 2014–January 2017	65 patients with cancer diagnosis, HSCT, and renal transplant recipients; primary prophylaxis	Q4W; 1–2 h; NA	NA	9/65 (13.8%). Lip/tongue/extremities tingling, dyspnea, and chest tightness. Nine patients discontinued IVP because of adverse events.
Savasan, USA, 2021, [32]	Retrospective, NA	25 patients received chemotherapy or HSCT	Q4W; 1 h; ondansetron	NA	22/25 (88%). Nasal congestion (n = 12), lip tingling (n = 8), nausea (n = 7), tongue tingling (n = 6), vomiting (n = 4), throat swelling (n = 4), throat tingling (n = 3), throat itching (n = 3), runny nose (n = 3), nose itching (n = 3), cough (n = 3), tongue swelling (n = 2), chest tightness (n = 2), lip swelling (n = 1), lip itching (n = 1), lip pain (n = 1), wheezing (n = 1), chest pain (n = 1), and skin rash (n = 1). No adverse events-related discontinuation.

Abbreviation: auto, autologous; allo, allogeneic; D, days; GVHD, graft versus host disease; HIV, human immunodeficiency virus; h, hours; HSCT, hematopoietic stem cell transplant; IVP, intravenous pentamidine; LFT, liver function test; M, months; NA, not available; PCP, *Pneumocystis* pneumonia; Q2W, every two weeks; Q3W, every three weeks; Q4W, every four weeks; SOT: solid organ transplantation. ^a^ Please refer to Section 2 for definition of “primary prophylaxis”, secondary prophylaxis”, “first-line prophylaxis”, and “second-line prophylaxis”. ^b^ The study only reported adverse reactions leading to discontinuation of IVP. This could be an underestimate of the incidence of adverse reactions in these studies.

## Data Availability

Not applicable.

## Supplementary Materials

The following supporting information can be downloaded at: https://www.mdpi.com/article/10.3390/jof9040406/s1. Figure S1: Forrest plot; Figure S2: Funnel plot; Table S1: PRISMA Checklist; Table S2: Keywords and search results in different databases; Table S3: Excluded studies and reasons; Table S4: Modified risk of bias tool developed By Hoy et al. and Edward et al; Table S5: Risk of bias analysis for all studies included in the review; Table S6: Sensitivity analysis and subgroup analysis of breakthrough PCP in patients who received intravenous pentamidine; Table S7: Meta-regression of breakthrough PCP in patients who received intravenous pentamidine; Table S8: Sensitivity analysis and subgroup analysis of incidence of adverse reaction in patients who received intravenous pentamidine; Table S9: Meta-regression of incidence of adverse reaction in patients who received intravenous pentamidine; Table S10: Sensitivity analysis and subgroup analysis of adverse event-related discontinuation in patients received intravenous pentamidine; Table S11: Meta-regression of adverse event-related discontinuation in patients received intravenous pentamidine.
