# Supplementary material for: Incidence of Pneumocystis Pneumonia in Immunocompromised Patients without Human Immunodeficiency Virus on Intravenous Pentamidine Prophylaxis: A Systematic Review and Meta-Analysis
Source: J Fungi (Basel). 2023 Mar 25;9(4):406. doi: 10.3390/jof9040406 (PMC10144649; doi:10.3390/jof9040406)

Supplementary documents

Table S1. PRISMA Checklist

Section and Topic	Item #	Checklist item	Location
**TITLE**			
Title	1	Identify the report as a systematic review.	Title
**ABSTRACT**			
Abstract	2	See the PRISMA 2020 for Abstracts checklist.	Methods
**INTRODUCTION**			
Rationale	3	Describe the rationale for the review in the context of existing knowledge.	Introduction
Objectives	4	Provide an explicit statement of the objective(s) or question(s) the review addresses.	Introduction
**METHODS**			
Eligibility criteria	5	Specify the inclusion and exclusion criteria for the review and how studies were grouped for the syntheses.	Methods
Information sources	6	Specify all databases, registers, websites, organisations, reference lists and other sources searched or consulted to identify studies. Specify the date when each source was last searched or consulted.	Methods
Search strategy	7	Present the full search strategies for all databases, registers and websites, including any filters and limits used.	Method,<br>Table S2
Selection process	8	Specify the methods used to decide whether a study met the inclusion criteria of the review, including how many reviewers screened each record and each report retrieved, whether they worked independently, and if applicable, details	Methods

Section and Topic	Item #	Checklist item	Location
		of automation tools used in the process.	
Data collection process	9	Specify the methods used to collect data from reports, including how many reviewers collected data from each report, whether they worked independently, any processes for obtaining or confirming data from study investigators, and if applicable, details of automation tools used in the process.	Methods, Table 1
Data items	10a	List and define all outcomes for which data were sought. Specify whether all results that were compatible with each outcome domain in each study were sought (e.g. for all measures, time points, analyses), and if not, the methods used to decide which results to collect.	Methods, Table 1
	10b	List and define all other variables for which data were sought (e.g. participant and intervention characteristics, funding sources). Describe any assumptions made about any missing or unclear information.	Methods , Table 1
Study risk of bias assessment	11	Specify the methods used to assess risk of bias in the included studies, including details of the tool(s) used, how many reviewers assessed each study and whether they worked independently, and if applicable, details of automation tools used in the process.	Methods
Effect measures	12	Specify for each outcome the effect measure(s) (e.g. risk ratio, mean difference) used in the synthesis or presentation of results.	Methods
Synthesis methods	13a	Describe the processes used to decide which studies were eligible for each synthesis (e.g. tabulating the study intervention characteristics and comparing against the planned groups for each synthesis (item #5)).	Methods, Figure S1, Table 1, Table S3
	13b	Describe any methods required to prepare the data for presentation or synthesis, such as handling of missing summary statistics, or data conversions.	Methods, Table 1
	13c	Describe any methods used to tabulate or visually display results of individual studies and syntheses.	Methods

Section and Topic	Item #	Checklist item	Location
	13d	Describe any methods used to synthesize results and provide a rationale for the choice(s). If meta-analysis was performed, describe the model(s), method(s) to identify the presence and extent of statistical heterogeneity, and software package(s) used.	Methods
	13e	Describe any methods used to explore possible causes of heterogeneity among study results (e.g. subgroup analysis, meta-regression).	Methods
	13f	Describe any sensitivity analyses conducted to assess robustness of the synthesized results.	Methods
Reporting bias assessment	14	Describe any methods used to assess risk of bias due to missing results in a synthesis (arising from reporting biases).	Methods
Certainty assessment	15	Describe any methods used to assess certainty (or confidence) in the body of evidence for an outcome.	Methods
**RESULTS**			
Study selection	16a	Describe the results of the search and selection process, from the number of records identified in the search to the number of studies included in the review, ideally using a flow diagram.	Results, Figure S1, Table S2-S3
	16b	Cite studies that might appear to meet the inclusion criteria, but which were excluded, and explain why they were excluded.	Results, Table S3
Study characteristics	17	Cite each included study and present its characteristics.	Results, Table 1
Risk of bias in studies	18	Present assessments of risk of bias for each included study.	Results, Table 1, Figure S3
Results of individual studies	19	For all outcomes, present, for each study: (a) summary statistics for each group (where appropriate) and (b) an effect estimate and its precision (e.g. confidence/credible interval), ideally using structured tables or plots.	Table 1, Figure S3

Section and Topic	Item #	Checklist item	Location
Results of syntheses	20a	For each synthesis, briefly summarise the characteristics and risk of bias among contributing studies.	Results
	20b	Present results of all statistical syntheses conducted. If meta-analysis was done, present for each the summary estimate and its precision (e.g. confidence/credible interval) and measures of statistical heterogeneity. If comparing groups, describe the direction of the effect.	Results
	20c	Present results of all investigations of possible causes of heterogeneity among study results.	Results, Table S4-S6
	20d	Present results of all sensitivity analyses conducted to assess the robustness of the synthesized results.	Results, Table S4-S6
Reporting biases	21	Present assessments of risk of bias due to missing results (arising from reporting biases) for each synthesis assessed.	Results
Certainty of evidence	22	Present assessments of certainty (or confidence) in the body of evidence for each outcome assessed.	Results
**DISCUSSION**			
Discussion	23a	Provide a general interpretation of the results in the context of other evidence.	Discussion
	23b	Discuss any limitations of the evidence included in the review.	Discussion
	23c	Discuss any limitations of the review processes used.	Discussion
	23d	Discuss implications of the results for practice, policy, and future research.	Discussion
**OTHER INFORMATION**			
Registration and protocol	24a	Provide registration information for the review, including register name and registration number, or state that the review was not registered.	Methods
	24b	Indicate where the review protocol can be accessed, or state that a protocol was not prepared.	Methods, Table S2-S3

Section and Topic	Item #	Checklist item	Location
	24c	Describe and explain any amendments to information provided at registration or in the protocol.	Methods,<br>Table S2-S3
Support	25	Describe sources of financial or non-financial support for the review, and the role of the funders or sponsors in the review.	Funding
Competing interests	26	Declare any competing interests of review authors.	Conflicts of Interest
Availability of data, code and other materials	27	Report which of the following are publicly available and where they can be found: template data collection forms; data extracted from included studies; data used for all analyses; analytic code; any other materials used in the review.	Results, Figure S1,<br>Table 1

Table S2. Keywords and search results in different databases

Database	Keyword	Filter	Date	Results
PubMed	(intravenous pentamidine) AND (pneumocystis pneumonia OR Pneumocystis jirovecii pneumonia) AND (prophylaxis)	NA	December, 15,2022	76
Embase	'intravenous pentamidine' AND ('pneumocystis pneumonia' OR 'pneumocystis jirovecii pneumonia') AND 'prophylaxis'	Title Abstract Keyword	December, 15,2022	44
Web of Science	(intravenous pentamidine) AND (pneumocystis pneumonia OR Pneumocystis jirovecii pneumonia) AND (prophylaxis)	NA	December 15, 2022	78
Cochrane Library	(intravenous pentamidine) AND (pneumocystis pneumonia OR Pneumocystis jirovecii pneumonia) AND (prophylaxis)	Title Abstract Keyword	December, 15,2022	6
ClinicalTrials.gov	(intravenous pentamidine) AND (pneumocystis pneumonia OR Pneumocystis jirovecii pneumonia) AND (prophylaxis)	Condition of disease	December, 15,2022	0

NA, not applied

Table S3. Excluded studies and reasons

Citations	Reasons
Mantadakis E. Pneumocystis jirovecii Pneumonia in Children with Hematological Malignancies: Diagnosis and Approaches to Management. J Fungi (Basel). 2020;6(4):331.	Review article
Diken AI, Diken OE, Hanedan O, et al. Pentamidine in Pneumocystis jirovecii prophylaxis in heart transplant recipients. World J Transplant. 2016;6(1):193-198.	Review article
Gupta M, Stephenson K, Gauar S, Frenkel L. Intravenous pentamidine as an alternate for Pneumocystis carinii pneumonia prophylaxis in children with HIV infection. Pediatr Pulmonol Suppl. 1997;16:199-200.	People living with HIV
Yeung KT, Chan M, Chan CK. The safety of i.v. pentamidine administered in an ambulatory setting. Chest. 1996;110(1):136-140. doi:10.1378/chest.110.1.136	People living with HIV
Ena J, Amador C, Pasquau F, et al. Once-a-month administration of intravenous pentamidine to patients infected with human immunodeficiency virus as prophylaxis for Pneumocystis carinii pneumonia. Clin Infect Dis. 1994;18(6):901-904.	People living with HIV
Fahy JV, Chin DP, Schnapp LM, et al. Effect of aerosolized pentamidine prophylaxis on the clinical severity and diagnosis of Pneumocystis carinii pneumonia. Am Rev Respir Dis. 1992;146(4):844-848.	Aerosolized pentamidine

Table S4. Modified risk of bias tool developed By Hoy et al. and Edward et al.

**External Validity**
1. Was the sampling frame a true or close representation of the target population?
2. Was some form of random selection used to select the sample, OR was a census undertaken?
3. Was the likelihood of nonresponse bias minimal?
**Internal Validity**
4. Were data collected directly from the subjects (as opposed to a proxy)?
5. Was an acceptable case definition used in the study?
6. Was the study instrument that measured the parameter of interest shown to have validity and reliability?
7. Was the same mode of data collection used for all subjects?
8. Was the length of the shortest prevalence period for the parameter of interest appropriate?
9. Were the numerator(s) and denominator(s) for the parameter of interest appropriate?
**Summary risk of bias**
10. Summary item on the overall risk of study bias

Notes: The modified tool assesses each study according to nine domains: three external validity domains, and six internal validity domains, plus one item assessing the overall risk of bias. The external validity domains assess the target population; sampling and non-response bias, while the internal risk of bias domains assess data collection, case definitions, assessment tools, prevalence period, and an assessment of the numerator and denominator. Items 1 to 9 were rated as either low (0 star) or high (1 star) risk, and item 10 (overall assessment) was rated as either low (1-3 stars), moderate (4-6 stars), or high risk (7-9 stars).

Table S5. Risk of bias analysis for all studies included in the review.

Study	1. Sampling frame represent target population?	2. Random selection for sample or census?	3. Likelihood of nonresponse bias minimal?	4. Data collected directly from subjects?	5. Acceptable case definition?	6. Study Instrument has validity and reliability?	7. Same mode of data collection for all subjects?	8. Length of prevalence period appropriate?	9. Appropriate numerator and denominator?	10. Overall Risk Assessment
Lim, USA, 2015	Low	High	Low	Low	High	Low	High	High	Low	Moderate
Diri, USA, 2016,	High	High	Low	Low	High	Low	Low	High	Low	Moderate
Sweiss, USA, 2018	High	Low	Low	Low	Low	Low	High	Low	Low	Low
Awad, Jorden, 2020	High	High	Low	Low	Low	Low	High	High	Low	Moderate
McCollam, USA,	Low	High	Low	Low	Low	Low	Low	High	Low	Low

2022										
Kim, USA, 2008	High	High	Low	Low	Low	Low	Low	High	Low	Low
Prasad, USA, 2008	High	High	Low	Low	Low	Low	Low	High	Low	Low
DeMasi, USA, 2013	High	High	Low	Low	Low	Low	Low	High	Low	Low
Orgel, USA, 2014	High	High	Low	Low	Low	Low	Low	High	Low	Low
Clark, USA, 2015	High	High	Low	Low	Low	Low	Low	High	Low	Low
Curi, USA, 2016,	Low	High	Low	Low	Low	Low	High	High	Low	Low
Levy, USA, 2016	High	High	Low	Low	Low	Low	Low	High	Low	Low

Solodokin, USA, 2016	High	High	Low	Low	Low	Low	Low	High	Low	Low
Tamyao, Spain, 2017	High	High	Low	High	Low	Low	Low	High	Low	Moderate
Kruizinga, Netherland, 2017	High	High	Low	Low	Low	Low	Low	High	Low	Low
Quinn, USA, 2018	High	High	Low	Low	Low	Low	Low	High	Low	Low
Brown, USA, 2020	High	High	Low	Low	Low	Low	High	High	Low	Moderate
Savasan, USA, 2021	High	High	Low	Low	Low	Low	High	High	Low	Moderate

Table S6. Sensitivity analysis and subgroup analysis of breakthrough PCP in patients who received intravenous pentamidine.

Analysis	Studies, n	Total patients, n	Incidence (95% CI)	I^2 heterogeneity
Model				
Random effects model	16	3,025	0.007 (0.003-0.014)	42.025
Fixed effects model	16	3,025	0.009 (0.005-0.015)	42.025
Age				
<18 years	11	1,874	0.008 (0.003-0.021)	52.221
≥18 years	5	1,151	0.003 (0.001-0.011)	0.000
Geographic region				
USA	13	2,677	0.007 (0.003-0.016)	52.207
Outside USA	3	348	0.007 (0.002-0.027)	0.000
Total patient number				
≤100	4	216	0.021 (0.003-0.142)	64.743
>100	12	2,809	0.005 (0.003-0.010)	0.000
Frequency of IVP				
Q4W	11	2,288	0.007 (0.002-0.020)	57.946
First-line therapy[a]	7	752	0.005 (0.002-0.014)	0.000

Abbreviation: CI, confidence interval; IVP, intravenous pentamidine; PCP, *Pneumocystis jirovecii* pneumonia; Q4W, every 4 weeks; USA, United States of America.

a. Total 8 studies had patients used IVP as first-line prophylaxis, but 1 article (Kruizinga, et al.) was excluded because the original data combined first-line prophylaxis and second-line prophylaxis. The meta-regression is not performed in this category because the original data are incomplete.

Table S7. Meta-regression of breakthrough PCP in patients who received intravenous pentamidine.

**Variable**[a]	**Single covariate**			**Multiple covariates**		
	Coefficient	95% CI	*p* value	Coefficient	95% CI	*p* value
Age group	0.988	-0.707 to 2.682	0.253	1.956	0.494 to 3.418	**0.009**
Geographic region	-0.048	-2.083 to 1.987	0.963	-0.224	-1.737 to 1.290	0.772
Total patient number	2.011	0.636 to 3.386	0.004	2.249	0.956 to 3.542	**0.001**
Frequency of IVP	-0.369	-2.026 to 1.288	0.662	-0.936	-2.224 to 0.352	0.154

Abbreviation: CI, confidence interval; IVP, intravenous pentamidine; PCP, *Pneumocystis jirovecii* pneumonia; Q4W, every 4 weeks; USA, United States of America

a. Reference groups are adult population, study in the USA, patient number >100, and IVP Q4W

Table S8. Sensitivity analysis and subgroup analysis of incidence of adverse reaction in patients who received intravenous pentamidine.

**Analysis**	**Studies, n**	**Total patients, n**	**Incidence (95% CI)**	***I^2* heterogeneity**
Model				
Random effects model	14	2,068	0.113 (0.067-0.186)	90.917
Fixed effects model	14	2,068	0.125 (0.108-0.144)	90.917
Age[a]				
<18 years	9	1,642	0.095 (0.059-0.149)	87.444
≥18 years	3	336	0.027 (0.001-0.518)	92.777
Geographic region				
USA	11	1,708	0.132 (0.073-0.228)	92.131
Outside USA	3	360	0.048 (0.008-0.236)	87.445
Total patient number				
≤100	5	294	0.189 (0.047-0.525)	91.829
>100	9	1,774	0.086 (0.051-0.142)	88.855
Frequency of IVP				
Q4W	9	1,331	0.123 (0.053-0.257)	93.466

Abbreviation: CI, confidence interval; IVP, intravenous pentamidine; Q4W, every 4 weeks; USA, United States of America.

a. Two studies (Brown, et al. and Savasan, et al.) combined pediatric and adult population, which are not included in this subgroup analysis

Table S9. Meta-regression of incidence of adverse reaction in patients who received intravenous pentamidine.

**Variable**[a]	**Single covariate**			**Multiple covariates**		
	Coefficient	95% CI	*p* value	Coefficient	95% CI	*p* value
Age group						
Adult	Reference			Reference		
Pediatric	0.558	-1.060 to 2.176	0.499	1.404	-1.176 to 3.984	0.286
Mixed	2.718	0.597 to 4.838	0.012	2.449	-0.155 to 5.052	0.065
Geographic region	-0.973	-2.525 to 0.579	0.219	-0.715	-2.474 to 1.044	0.426
Total patient number	1.118	-0.077 to 2.313	0.067	0.961	-1.316 to 3.237	0.408
Frequency of IVP	-0.382	-1.601 to 0.838	0.540	-0.275	-1.935 to 1.386	0.746

Abbreviation: CI, confidence interval; IVP, intravenous pentamidine; Q4W, every 4 weeks; USA, United States of America

a. Reference groups are adult population, study in the USA, patient number >100, and IVP Q4W

Table S10. Sensitivity analysis and subgroup analysis of adverse event-related discontinuation in patients received intravenous pentamidine.

**Analysis**	**Studies, n**	**Total patients, n**	**Incidence (95% CI)**	**I^2 heterogeneity**
Model				
Random effects model	11	1,802	0.037 (0.018-0.073)	84.737
Fixed effects model	11	1,802	0.065 (0.052-0.081)	84.737
Age[a]				
<18 years	8	1,525	0.036 (0.016-0.077)	86.966
≥18 years	N/A[b]			
Geographic region				
USA	8	1,442	0.047 (0.021-0.099)	87.305
Outside USA	3	360	0.018 (0.006-0.055)	29.663
Total patient number				
≤100	3	145	0.066 (0.019-0.202)	58.150
>100	8	1,657	0.030 (0.012-0.069)	87.975
Frequency of IVP				
Q4W	7	1,182	0.020 (0.007-0.057)	79.148

Abbreviation: CI, confidence interval; IVP, intravenous pentamidine; N/A, not applicable; Q4W, every 4 weeks; USA, United States of America.

a. Two studies (Brown, et al. and Savasan, et al.) combined pediatric and adult populations, which are not included in this subgroup analysis.

b. Only one publication (Awad, et al.) in this subgroup.

Table S11. Meta-regression of adverse event-related discontinuation in patients received intravenous pentamidine.

**Variable**[a]	**Single covariate**			**Multiple covariates**		
	Coefficient	95% CI	*p* value	Coefficient	95% CI	*p* value
Age group						
Adult	Reference			Reference		
Pediatric	2.627	-0.903 to 6.158	0.145	1.867	-1.929 to 5.662	0.335
Mixed	3.515	-0.370 to 7.400	0.076	4.800	-1.578 to 11.177	0.140
Geographic region	-1.105	-2.809 to 0.598	0.203	0.146	-2.255 to 2.547	0.905
Total patient number	0.750	-1.002 to 2.501	0.402	-1.017	-4.342 to 2.308	0.549
Frequency of IVP	1.224	-0.188 to 2.635	0.089	1.800	0.265 to 3.335	**0.022**

Abbreviation: CI, confidence interval; IVP, intravenous pentamidine; Q4W, every 4 weeks; USA, United States of America

a. Reference groups are adult population, study in the USA, patient number >100, and IVP Q4W

Supplemental Figure 1.

A. Forest plot of breakthrough *Pneumocystis jirovecii* pneumonia in patients who received intravenous pentamidine.

Study name	Statistics for each study			Relative weight
	Event rate	Lower limit	Upper limit	
Kim, 2008	0.013	0.004	0.039	11.74
Prasad, 2008	0.167	0.042	0.477	9.72
DeMasi, 2013	0.004	0.000	0.055	5.05
Orgel, 2014	0.009	0.001	0.058	7.66
Clark, 2015	0.003	0.000	0.021	7.68
Lim, 2015	0.005	0.000	0.075	5.05
Curi, 2016	0.003	0.000	0.053	5.05
Levy, 2016	0.004	0.000	0.067	5.05
Solodokin, 2016	0.004	0.000	0.062	5.05
Diri, 2016	0.004	0.000	0.066	5.05
Tamyao, 2017	0.009	0.001	0.127	5.03
Kruizinga, 2017	0.009	0.001	0.064	7.66
Quinn, 2018	0.001	0.000	0.015	5.06
Swiss, 2018	0.010	0.001	0.138	5.03
Awad, 2020	0.003	0.000	0.041	5.06
McCollam, 2022	0.001	0.000	0.011	5.06
	0.007	0.003	0.014	

Event rate and 95% CI

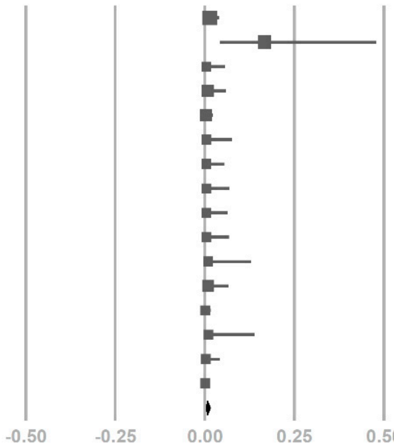

B. Forest plot of incidence of adverse reactions in patients who received intravenous pentamidine.

Study name	Statistics for each study			Relative weight
	Event rate	Lower limit	Upper limit	
DeMasi, 2013	0.088	0.050	0.148	7.93
Orgel, 2014	0.085	0.047	0.152	7.80
Clark, 2015	0.060	0.039	0.091	8.22
Lim, 2015	0.005	0.000	0.075	2.88
Curi, 2016	0.204	0.146	0.278	8.29
Levy, 2016	0.126	0.076	0.202	8.00
Solodokin, 2016	0.157	0.102	0.233	8.15
Tamyao, 2017	0.055	0.018	0.156	6.40
Kruizinga, 2017	0.178	0.119	0.258	8.18
Quinn, 2018	0.022	0.012	0.039	7.92
Swiss, 2018	0.340	0.223	0.480	7.95
Awad, 2020	0.005	0.001	0.037	4.32
Brown, 2020	0.138	0.074	0.245	7.67
Suvasan, 2021	0.880	0.687	0.961	6.28
	0.113	0.067	0.186	

Event rate and 95% CI

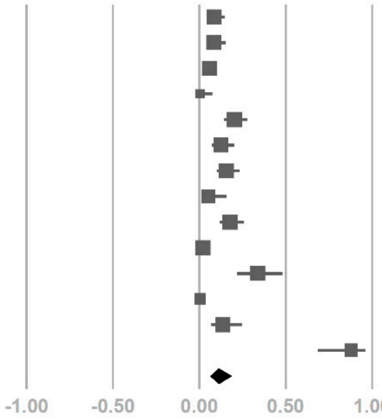

C. Forest plot of intravenous pentamidine discontinuation due to adverse events.

Study name	Statistics for each study			Relative weight
	Event rate	Lower limit	Upper limit	
DeMasi, 2013	0.015	0.004	0.056	8.87
Clark, 2015	0.060	0.039	0.091	12.58
Curi, 2016	0.003	0.000	0.053	4.50
Levy, 2016	0.189	0.127	0.273	12.51
Solodokin, 2016	0.041	0.017	0.095	11.01
Tamyao, 2017	0.036	0.009	0.134	8.81
Kruizinga, 2017	0.017	0.004	0.065	8.87
Quinn, 2018	0.022	0.012	0.039	12.14
Awad, 2020	0.003	0.000	0.041	4.50
Brown, 2020	0.138	0.074	0.245	11.76
Suvasan, 2021	0.019	0.001	0.244	4.45
	0.037	0.018	0.073	

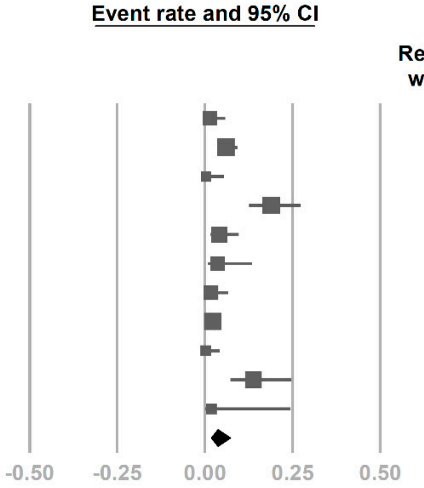

Supplementary Figure 2.

The following figures show the funnel plots of the studies.

A. Funnel plot of the studies evaluating breakthrough *Pneumocystis jirovecii* pneumonia in patients who received intravenous pentamidine.
   The funnel plot of included trials showed asymmetric distribution. The *p* value of the Egger's test was 0.013, indicating potential publication bias.

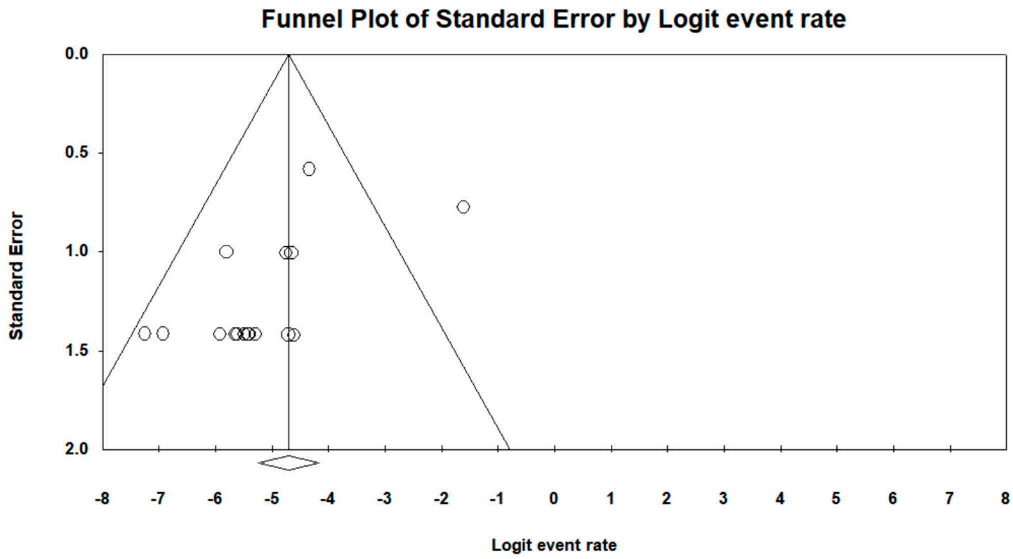

B. Funnel plot of the studies evaluating the Incidence of adverse reactions in patients who received intravenous pentamidine.
   The *p* value of the Egger's test was 0.594.

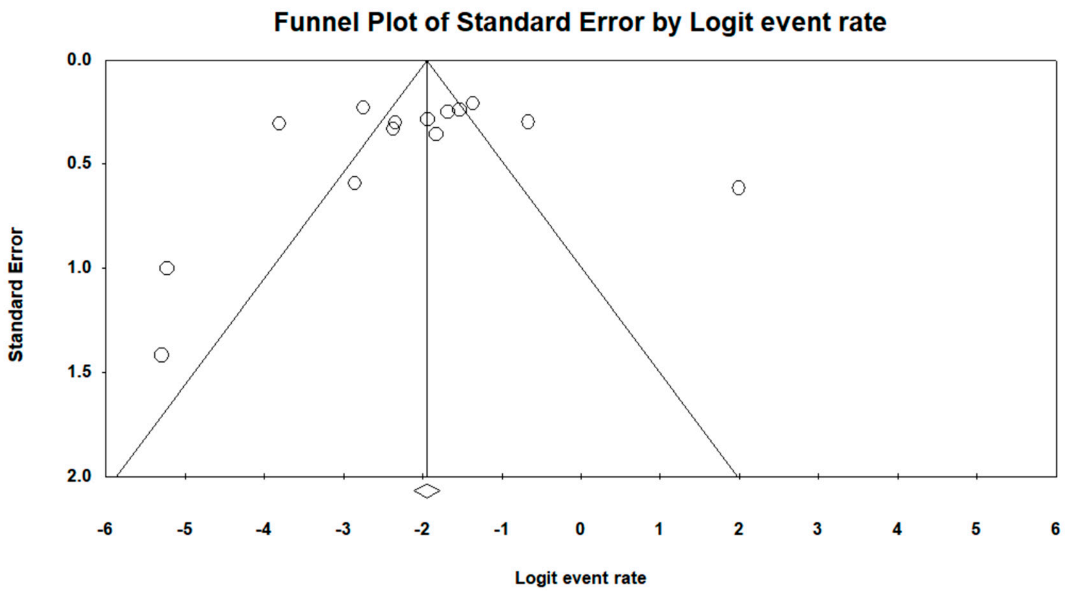

C. Funnel plot of the studies evaluating discontinuation of intravenous pentamidine due to adverse events

The *p* value of the Egger's test was 0.061.

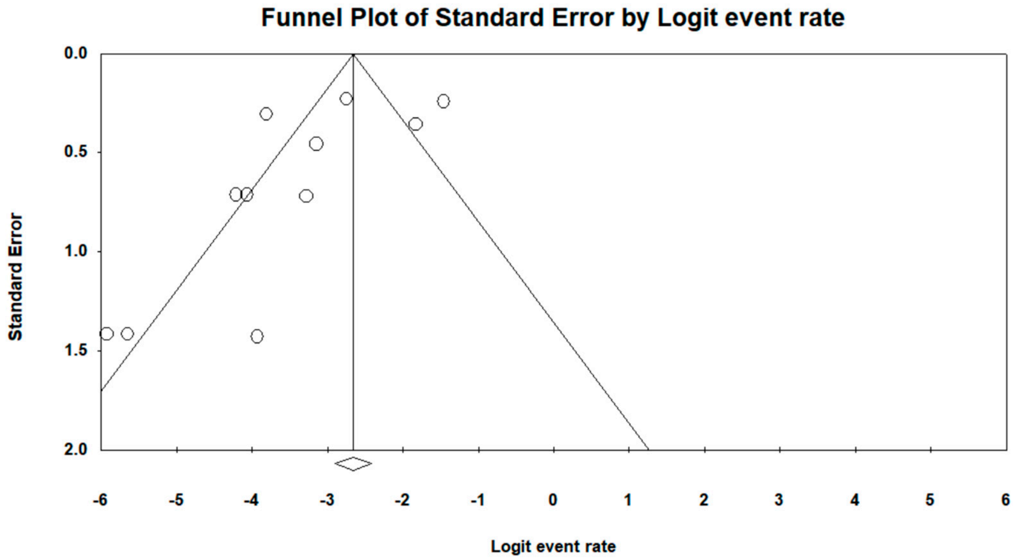

Supplement: Supplementary file 1 [file jof-09-00406-s001.zip › jof-2250301-supplementary.pdf]
